# Use of a new proximity assay (NanoBRET) to investigate the ligand‐binding characteristics of three fluorescent ligands to the human *β*_1_‐adrenoceptor expressed in HEK‐293 cells

**DOI:** 10.1002/prp2.250

**Published:** 2016-08-08

**Authors:** Mark Soave, Leigh A. Stoddart, Alastair Brown, Jeanette Woolard, Stephen J. Hill

**Affiliations:** ^1^ Cell Signalling and Pharmacology Research GroupSchool of Life SciencesUniversity of NottinghamNottinghamNG7 2UHUnited Kingdom; ^2^ Heptares Therapeutics Ltd.Bio ParkWelwyn Garden CityAL7 3AXUnited Kingdom

**Keywords:** Bioluminescence energy transfer, ligand binding, probe dependence, *β*‐adrenoceptors

## Abstract

Previous research has indicated that allosteric interactions across the dimer interface of *β*_1_‐adrenoceptors may be responsible for a secondary low affinity binding conformation. Here we have investigated the potential for probe dependence, in the determination of antagonist pK_i_ values at the human *β*_1_‐adenoceptor, which may result from such allosterism interactions. Three fluorescent *β*_1_‐adrenoceptor ligands were used to investigate this using bioluminescence energy transfer (BRET) between the receptor‐bound fluorescent ligand and the N‐terminal NanoLuc tag of a human *β*_1_‐adrenoceptor expressed in HEK 293 cells (NanoBRET). This proximity assay showed high‐affinity‐specific binding to the NanoLuc‐ *β*_1_‐adrenoceptor with each of the three fluorescent ligands yielding *K*_D_ values of 87.1 ± 10 nmol/L (*n* = 8), 38.1 ± 12 nmol/L (*n* = 7), 13.4 ± 2 nmol/L (*n* = 14) for propranolol‐Peg8‐BY630, propranolol‐ *β*(Ala‐Ala)‐BY630 and CGP‐12177‐TMR, respectively. Parallel radioligand‐binding studies with ^3^H‐CGP12177 and TIRF microscopy, to monitor NanoLuc bioluminescence, confirmed a high cell surface expression of the NanoLuc‐ *β*_1_‐adrenoceptor in HEK 293 cells (circa 1500 fmol.mg protein^−1^). Following a 1 h incubation with fluorescent ligands and *β*_1_‐adrenoceptor competing antagonists, there were significant differences (*P* < 0.001) in the pK_i_ values obtained for CGP20712a and CGP 12177 with the different fluorescent ligands and ^3^H‐CGP 12177. However, increasing the incubation time to 2 h removed these significant differences. The data obtained show that the NanoBRET assay can be applied successfully to study ligand‐receptor interactions at the human *β*_1_‐adrenoceptor. However, the study also emphasizes the importance of ensuring that both the fluorescent and competing ligands are in true equilibrium before interpretations regarding probe dependence can be made.

**Abbreviations**
BRET: bioluminescence energy transfer
DMEM: Dulbecco's modified Eagles medium
FCS: fetal calf serum
HEK: human embryonic kidney
Nluc: NanoLuc
PBS: phosphate‐buffered saline

## Introduction

The human *β*_1_‐adrenoceptor appears to exist in two active conformations (Kaumann et al. 2001; Kaumann and Molenaar 2008; Pak and Fishman 1996; Granneman 2001; Baker et al. 2003). One of these is a classical orthosteric‐binding site via which endogenous ligands, such as adrenaline and noradrenaline, mediate their agonist effects. The actions of agonists at this site are potently and competitively antagonized by *β*‐blockers such as CGP 20712A, CGP 12177, and propranolol (Baker et al. 2003; Baker 2005; Lowe et al. 2002; Joseph et al. 2004a). However, at much higher concentrations than required to occupy the orthosteric site, CGP 12177 (Staehelin et al. 1983) is able to produce an agonist response that is effectively resistant to *β*‐blocker antagonism at the concentrations normally employed to prevent the binding of agonists to the orthosteric site (Pak and Fishman 1996; Baker et al. 2003; Baker 2005; Joseph et al. 2004a; Konkar et al. 2000). The dissociation constants of classical *β*‐adrenoceptor antagonists are therefore one to two orders of magnitude higher when determined from inhibition of functional responses mediated by CGP 12177 at the *β*_1_‐adrenoceptor compared to those determined from antagonism of catecholamine responses (Molenaar et al. 2007; Lowe et al. 2002; Baker et al. 2003; Baker 2005). Studies in recombinant cell systems and cardiac tissue isolated from *β*_2_‐ and *β*_1_‐/*β*_2_‐adrenoceptor knockout mice have confirmed that the second‐site pharmacology of CGP 12177 is a direct consequence of its interaction with the *β*_1_‐adrenoceptor (Kaumann et al. 2001; Kaumann and Molenaar 2008; Pak and Fishman 1996).

Site‐directed mutagenesis studies have attempted to isolate the regions of the *β*_1_‐adrenoceptor responsible for this second site pharmacology in CHO‐K1 cells (Baker et al. 2008, 2014; Joseph et al. 2004b). Baker et al. (2014) have indicated that residues within the dimer interface region in transmembrane domain (TM) 4 of the *β*_1_‐adrenoceptor may be responsible for the secondary *β*_1_‐adrenoceptor conformation (Gherbi et al. 2015). In this situation, negative cooperativity across the TM4‐TM5 *β*_1_‐adrenoceptor homodimer interface may be responsible for generating the low affinity pharmacology of the secondary *β*_1_‐adrenoceptor conformation (Gherbi et al. 2015) in a manner analogous to that observed for the adenosine A_3_‐receptor (May et al. 2011; Corriden et al. 2014).

Recent studies with a fluorescent analog of CGP 12177 (BODIPY‐TMR‐CGP) have shown that at high concentrations it can begin to label both conformations of the human *β*_1_‐adrenoceptor expressed in CHO‐K1 cells (Gherbi et al. 2014, 2015). The availability of this ligand has made it possible to study the dynamics of ligand‐receptor interactions in living cells and provided evidence for negatively cooperative interactions (Gherbi et al. 2014, 2015). Recently, we developed an exquisitely sensitive proximity‐based method to monitor ligand‐receptor interactions in living cells using bioluminescence resonance energy transfer (BRET) (Stoddart et al. 2015). This used a recently described and extremely bright luciferase NanoLuc (Hall et al. 2012) fused to the N‐terminus of different GPCRs expressed in HEK 293 cells in conjunction with red‐shifted fluorescent ligands (Stoddart et al. 2015). This NanoBRET approach was able to show binding of fluorescent ligands to GPCRs in a highly specific way. Furthermore, in the case of the adenosine A_3_‐receptor where a number of different fluorescent ligands were available to probe this receptor, the study highlighted the fact that binding affinities varied depending on the A_3_‐receptor fluorescent probe used (Stoddart et al. 2015). These observations were in keeping with the known allosterism observed across the A_3_‐receptor homodimer interface (May et al. 2011; Corriden et al. 2014; Stoddart et al. 2015), and consistent with the expected probe dependence for such allosteric interactions (May et al. 2007, 2010; Christopoulos and Kenakin 2002).

Here, we have investigated the potential for probe dependence in the determination of antagonist pKi values at the human *β*_1_‐adenoceptor using three different fluorescent *β*_1_‐adrenoceptor ligands, in conjunction with a N‐terminal tagged NanoLuc human *β*_1_‐adrenoceptor expressed in HEK 293 cells.

## Materials and Methods

### cDNA constructs

To create the NL‐ *β*_1_‐AR construct, NanoLuc (NL) was initially ligated into pcDNA3.1 containing the 5‐HT receptor membrane localization signal sequence (sig) using KpnI and BamHI restriction enzymes, generating the sig‐NL plasmid. The *β*_1_ adrenoceptor with no start codon was then ligated to the C‐terminus of NL from pcDNA3.1 containing SNAP‐*β*_1_ (Gherbi et al. 2015) using BamHI and XbaI restriction enzymes. The resulting fusion protein contained a Gly‐Ser linker between the Nluc open reading frame (ORF) and the *β*_1_ ORF. The NanoLuc Histamine 1 receptor construct (NL‐H_1_) was made by removing the internal BamHI site in the H_1_ receptor (in pcDNA3.1) by site‐directed mutagenesis while maintaining the amino acid sequence. This was then ligated into the sig‐NL plasmid with BamHI and XhoI restriction enzymes.

### Materials

Propranolol‐Peg8‐BY630 (propranolol‐peg8‐BODIPY630/650) and prop‐*β*(Ala‐Ala)‐BY630 (propranolol‐*β*alanine‐ *β*alanine‐X‐BODIPY630/650) were obtained from CellAura (Nottingham, UK). BODIPY‐TMR‐CGP (CGP‐12177‐TMR) was purchased from Molecular Probes (Eugene, OR). Propranolol, ICI 118551, CGP 12177, CGP 20712A, and cimaterol were from Tocris (Bristol, UK). Isoprenaline was purchased from Sigma‐Aldrich (Gillingham, UK). The NanoLuc substrate furimazine was obtained from Promega (Southampton, UK). The radioligand ^3^H CGP 12177 was obtained from PerkinElmer (Coventry, UK).

### Cell lines

HEK 293 cells were maintained in Dulbecco's modified eagle medium (DMEM) containing 10% fetal calf serum (FCS) and 2 mmol/L l‐glutamine at 37°C in 5% CO_2_ atmosphere. Mixed population NL‐*β*_1_‐AR and NL‐H_1_ HEK 293 cell lines were generated using Fugene HD (Promega) according to the manufacturer's instructions and cells were then subjected to 1 mg/mL G418‐selective pressure for 2 weeks.

### Radioligand‐binding assays

Saturation‐ and competition‐binding assays were performed on stably transfected HEK NL‐ *β*_1_‐AR cells. These were seeded 24 h before experimentation in white Thermo Scientific 96‐well microplates. The medium was removed from each well and replaced with serum‐free media (DMEM with 2 mmol/L l‐glutamine), followed immediately by the required concentrations of ^3^H‐CGP 12177 radioligand and competing compounds. For saturation‐binding assays, nonspecific binding was determined in the presence of 10 *μ*mol/L propranolol. The cells were incubated for 2 h at 37°C, 5% CO_2_. The cells were washed twice by the addition and subsequent removal of 200 *μ*L phosphate‐buffered saline (PBS). 200 *μ*L microscint‐20 (PerkinElmer) was added to each well, a white base applied to the plate, and the plate sealed with a clear topseal heat seal (PerkinElmer). The plates were counted on a Topcount scintillation reader (PerkinElmer). Protein content of wells was determined by the method of Lowry et al. (1951).

### BRET NL‐*β*_1_‐AR receptor‐ligand‐binding assays

Saturation‐ and competition‐binding assays were performed on stably transfected HEK 293 cells. Cells were seeded 24 h before experimentation in white Thermo Scientific 96‐well microplates. The medium was removed from each well and replaced with HEPES‐buffered saline solution (HBSS; 147 mmol/L NaCL, 24 mmol/L KCl, 1.3 mmol/L CaCl_2_, 1 mmol/L MgSO_4_, 10 mmol/L HEPES, 2 mmol/L sodium pyruvate, 1.43 mmol/L NaHCO_3_, 4.5 mmol/L d‐glucose, pH 7.2–7.45) with the relevant concentration of fluorescent ligand and, if necessary, competing ligand. Nonspecific binding was determined with 10 *μ*mol/L propranolol. Cells were then incubated in the dark for 1 or 2 h at 37°C (no CO_2_). The NanoLuc substrate furimazine (Promega) was then added to each well at a final concentration of 10 *μ*mol/L and allowed to equilibrate for 5 min prior to reading. We measured the luminescence signals at two different wavelengths using the PHERAstar FS plate reader (BMG Labtech, UK) at room temperature. The filtered light from each well was simultaneously measured using 460 nm (80‐nm bandpass) and >610 nm longpass filters. The resulting raw BRET ratio was calculated by dividing the >610 nm emission by the 460 nm emission.

### Bioluminescence imaging of NL‐*β*_1_‐AR

Bioluniescence imaging experiments were performed to determine the cellular localization of the NL‐*β*_1_‐AR fusion protein. Imaging was performed using the Zeiss TIRF3 microscope equipped with a Photometrics Quantum EM camera and a 20× EC Plan‐Neofluar objective lens. No filter was used in these experiments, in order to maximize the luminescence detected by the camera. HEK 293 cells were seeded onto glass‐bottom MatTek dishes (Ashland, MA) and transiently transfected with the NL‐*β*_1_‐AR construct 24 h before experimentation using Fugene HD (Promega). The medium was removed and replaced with HBSS at 37°C. The luminescence of NL‐*β*_1_‐AR was captured as a single 10 sec exposure immediately after the addition of 10 *μ*mol/L furimazine. Following this a phase contrast image was acquired of the cells with a 33 msec exposure time. All images were acquired using the Zeiss Zen 2 software (Jena, Germany). Image processing was performed with FIJI software (Schindelin et al., 2012).

### Data analysis

Data were presented and analyzed using Prism 6 software (GraphPad).

Saturation‐binding curves were simultaneously fitted to obtain the total and nonspecific components using the following equation:

$$Radioligand\;binding\;or\;BRET\;ratio=\frac{B_{\max}\times \left[B\right]}{\left[B\right]+K_{D}}+\left(\left(M\times \left[B\right]\right)+C\right)$$

where *B*_max_ is the maximal level of specific binding, [B] is the concentration of fluorescent ligand or radioligand in nmol/L, *K*_D_ is the equilibrium dissociation constant in nmol/L, *M* is the slope of the linear nonspecific binding component, and C is the *y*‐axis intercept.

Competition radioligand and NanoBRET data were fitted using a one‐site sigmoidal response curve given by the following equation:

$$\%\;uninhibited\;binding=100-\frac{\left(100\times \left[A^{n}\right]\right)}{\left(\left[A^{n}\right]+IC_{50}^{n}\right)}+NS$$

where [A] is the concentration of competing drug, NS is the nonspecific binding, *n* is the Hill coefficient, and *IC*_50_ is the concentration of ligand required to inhibit 50% of the specific binding of the radioligand or fluorescent ligand.

The IC_50_ values from competition‐binding curves were used to calculate the *K*_i_ of the unlabeled ligands using the Cheng–Prusoff equation:

$$K_{i}=\frac{IC_{50}}{1+\frac{\left[L\right]}{K_{D}}}$$

where [*L*] is the concentration of fluorescent ligand or radioligand in nmol/L, and *K*_D_ is the dissociation constant of the fluorescent ligand or radioligand in nmol/L. The *K*_D_ values used were obtained from the saturation‐binding experiments.

Statistical significance was determined by one‐way analysis of variance (ANOVA, *P* < 0.05 considered significant).

## Results

### ^3^H‐CGP 12177‐binding experiments in NL‐*β*_1_‐AR HEK 293 cells

**Figure 1:**
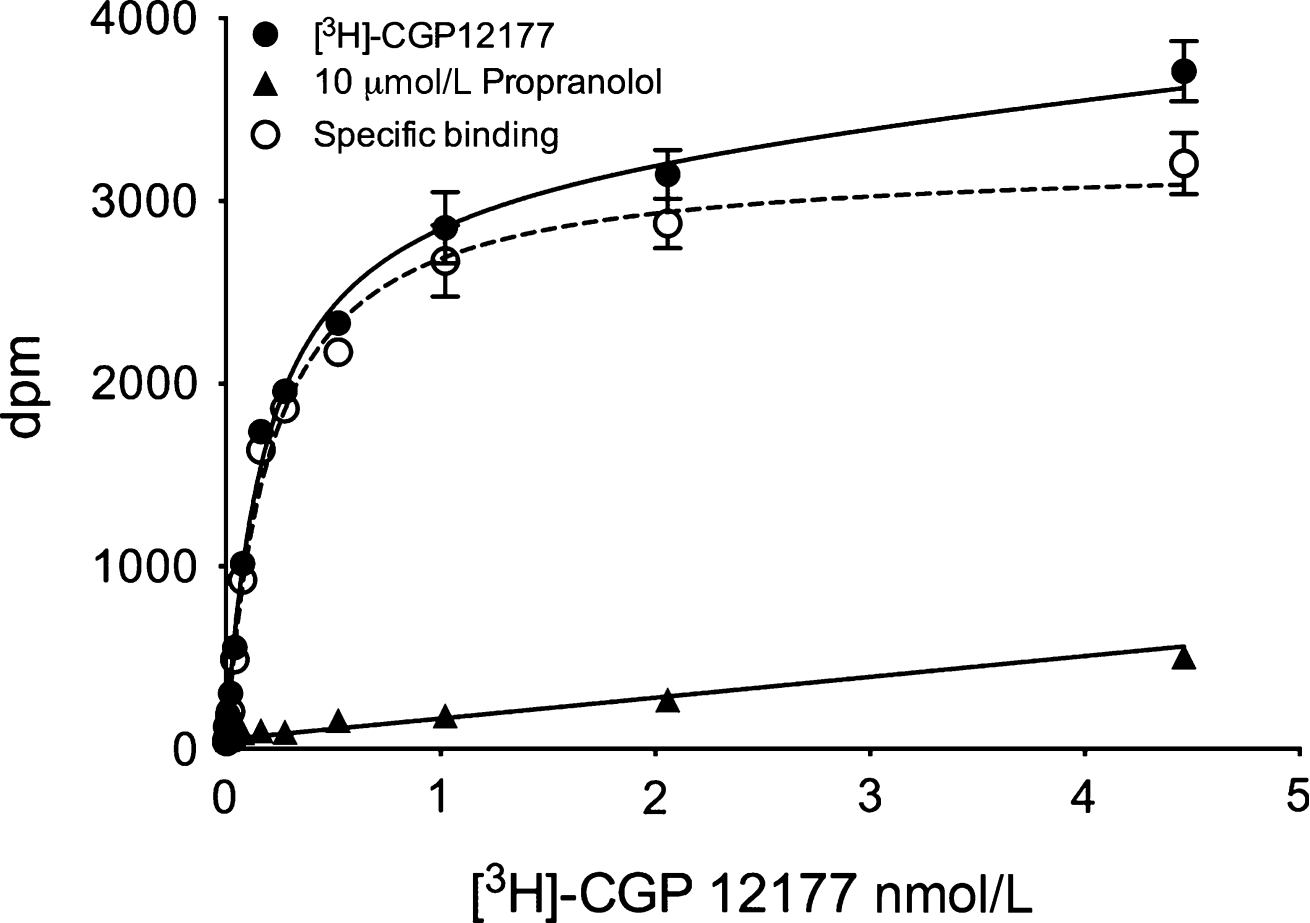
^3^H‐CGP 12177 binding to HEK‐NL‐*β*_1_‐AR cells showing total ^3^H‐CGP 12177 binding, nonspecific binding (obtained in the presence of 10 *μ*mol/L propranolol) and specific binding. Data points are mean ± SEM of quadruplicate determinations from a single experiment. Similar data were obtained in six separate experiments.

**Figure 2:**
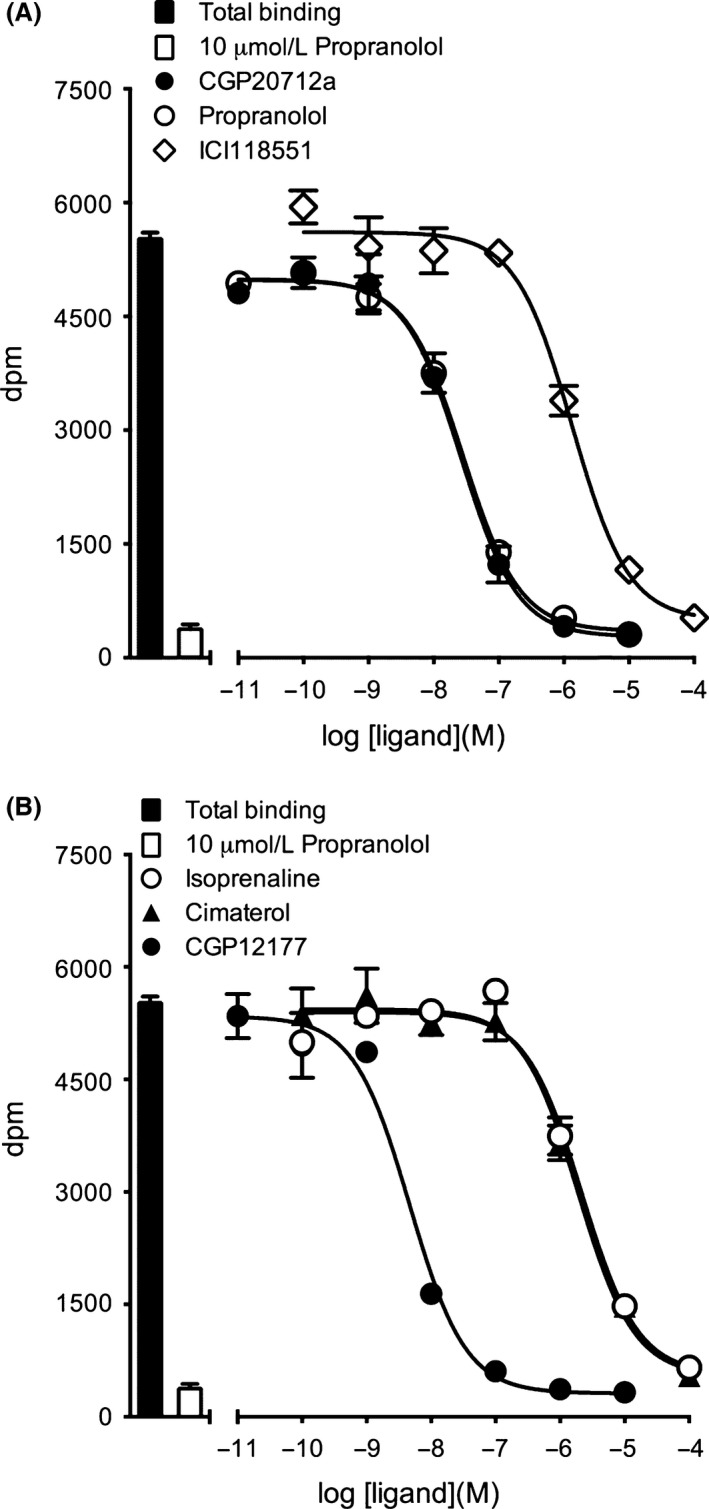
Inhibition of ^3^H‐CGP 12177 binding to HEK‐NL‐*β*_1_‐AR cells by (A) CGP 20712A, propranolol, ICI 118551, (B) isoprenaline, cimaterol, CGP 12177. Nonspecific binding was defined in the presence of 10 *μ*mol/L propranolol. The concentration of ^3^H‐CGP 12177 present in these experiments was 2.2 nmol/L. Data points are triplicate determinations of mean ± SEM and these single experiments are representative of five separate experiments. Summary data from the replicate experiments are provided in Table 1.

Initial radioligand‐binding experiments were performed with the high affinity ligand ^3^H‐CGP 12177 in HEK 293 cells stably expressing human *β*_1_‐adrenoceptors containing an N‐terminal Nanoluc luciferase fusion (NL‐*β*_1_‐ARs). Saturation analysis showed clear specific binding (Fig. 1; *K*_D_ for ^3^H‐CGP 12177 = 0.92 ± 0.19 nmol/L; *n* = 6) and a mean receptor expression level of 1581 ± 350 pmol.mg protein^−1^ (*n* = 6). The level of nonspecific binding deduced in the presence of 10 *μ*mol/L propranolol was low across the concentrations of ^3^H‐CGP 12177 employed (0–5 nmol/L; Fig. 1). Competition experiments with a range of *β*_1_‐adrenoceptor agonists and antagonists (Fig. 2A and B) fitted well to a simple single mass action equilibrium model and allowed the calculation of pK_i_ values for the different inhibitors (Table 1). As expected, the selective *β*_1_‐adrenoceptor antagonist CGP 20712A showed a much higher affinity for the NL‐*β*_1_‐adrenoceptor (pK_i_: 7.92; Fig. 2A; Table 1) than the *β*_2_‐adrenoceptor‐selective antagonist ICI 118551 (pK_i_ 6.01; Table 1; Fig. 2A), although both values were lower than those (8.81 and 6.52 for CGP 20712A and ICI 118551, respectively) obtained in CHO cells (Baker 2005).

**Table 1:** Binding affinities of competing ligands determined from inhibition of the specific binding of 0.9–2.5 nmol/L ^3^H‐CGP 12177, 100 nmol/L Propranolol‐Peg8‐BY630, 50 nmol/L Propranolol‐*β*(Ala‐Ala)‐BY630 or 50 nmol/L CGP‐12177‐TMR in HEK 293 cells expressing a NanoLuc‐tagged human *β*_1_‐adrenoceptor

	^3^H‐CGP‐12177		Propranolol‐Peg8‐BY630		Propranolol‐ *β*(Ala‐Ala)‐BY630		CGP‐12177‐TMR	
	pK_i_	*n*	pK_i_	*n*	pK_i_	*n*	pK_i_	*n*
Isoprenaline**	6.18 ± 0.07	5	6.72 ± 0.12	7	5.95 ± 0.09	8	6.56 ± 0.05	5
Cimaterol*	6.03 ± 0.05	5	6.44 ± 0.11	7	5.84 ± 0.22	8	6.34 ± 0.19	5
CGP 12177**	8.76 ± 0.09	5	8.67 ± 0.05	7	8.15 ± 0.10	8	8.93 ± 0.07	5
CGP 20712A**	7.92 ± 0.11	5	8.52 ± 0.09	7	7.84 ± 0.07	8	8.85 ± 0.19	5
Propranolol	7.92 ± 0.09	5	8.11 ± 0.12	5	7.77 ± 0.21	5	7.80 ± 0.08	5
ICI 118551	6.01 ± 0.13	5	6.55 ± 0.19	5	6.18 ± 0.22	5	6.51 ± 0.05	5

Incubations were for 1 h. Values show mean ± SEM obtained in *n* separate experiments. In each individual experiment triplicate determinations were made for each experimental condition. pKi values were determined from *IC*_50_ values using the Cheng–Prusoff equation as described under Methods. **,*pKi values obtained of competing ligand significantly differ between fluorescent/radioactive probes (**P < 0.001; *P < 0.05; One‐way ANOVA).

### Inhibition of ^3^H‐CGP 12177 binding by three different fluorescent *β*_1_‐adrenoceptor antagonists at the human *β*_1_‐AR

**Figure 3:**
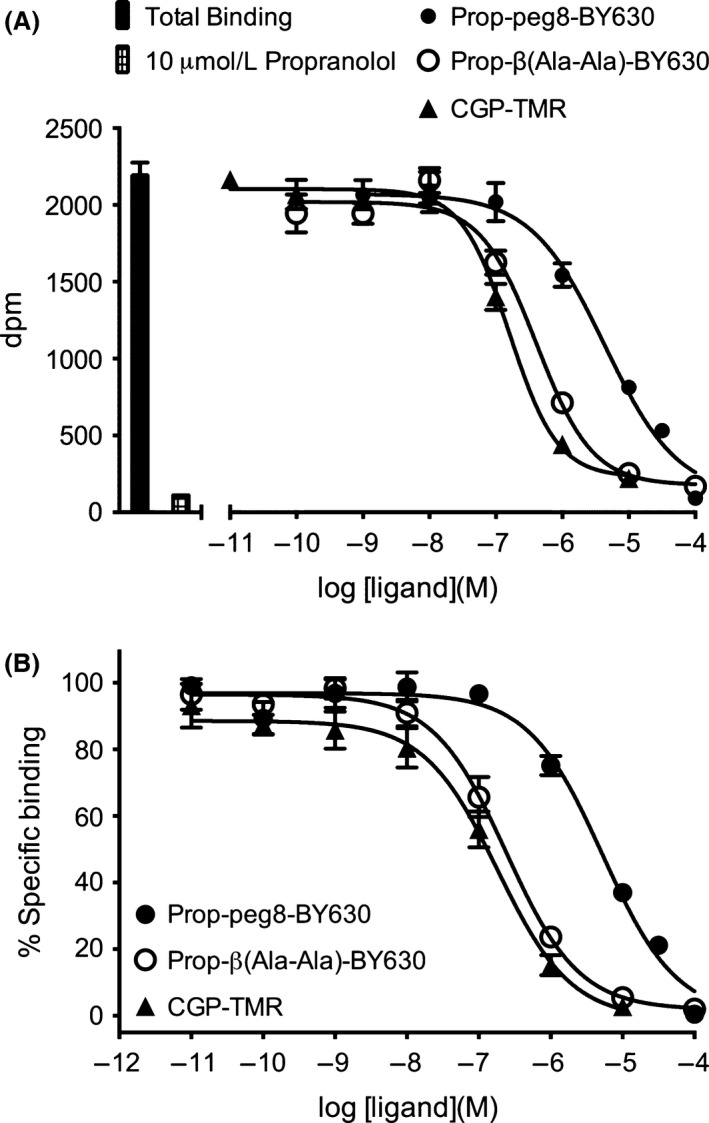
Inhibition of ^3^H‐CGP 12177 binding to HEK‐NL‐*β*_1_‐AR cells by propranolol‐Peg8‐BY630, propranolol‐*β*(Ala‐Ala)‐BY630 and CGP‐12177‐TMR. Nonspecific binding was defined in the presence of 10 *μ*mol/L propranolol. The concentration of ^3^H‐CGP 12177 present in these experiments was 0.9–2.5 nmol/L. Data points in (A) are mean ± SEM of triplicate determinations in a single representative experiment. (B) Inhibition of specific ^3^H‐CGP 12177 binding by each fluorescent ligand expressed as a percentage of the specific binding obtained in the absence of inhibitor. Data points in (B) are mean ± SEM of six (propranolol‐Peg8‐BY630), seven (propranolol‐*β* (Ala‐Ala)‐BY630), and five (CGP‐12177‐TMR) separate experiments. Summary data from the replicate experiments are provided in Table 2.

Three different fluorescent *β*‐AR ligands were used to investigate binding to the *β*_1_‐AR in this study: (1) Propranolol‐Peg8‐BY630 (Baker et al. 2011); (2) Propranolol‐ *β*(Ala‐Ala)‐BY630 (Stoddart et al. 2015), and (3) CGP‐12177‐TMR (Gherbi et al. 2014). Initial studies were performed to evaluate the ability of these ligands to inhibit the binding of ^3^H‐CGP 12177 (Fig. 3 and Table 2). The pK_i_ values obtained from these studies indicated that Propranolol‐*β*(Ala‐Ala)‐BY630 and CGP‐12177‐TMR were approximately an order of magnitude higher affinity than Propranolol‐Peg8‐BY630 in HEK‐293 cell expressing the human *β*_1_‐adrenoceptor (Table 2).

**Table 2:** Binding affinities of three fluorescent ligands determined from inhibition of the specific binding of 0.9–2.5 nmol/L ^3^H‐CGP 12177 and from the saturation of Prop‐peg8‐BY630, Prop‐ *β*(Ala‐Ala)‐BY630, or CGP‐12177‐TMR in HEK 293 cells expressing a NanoLuc‐tagged human *β*_1_‐adrenoceptor

	Radioligand		1 h NanoBRET		2 h NanoBRET	
Fluorescent Ligand	pK_i_	*n*	pK_d_	*n*	pK_d_	*n*
Prop‐peg8‐BY630	6.17 ± 0.15	6	7.06 ± 0.12	8	6.18 ± 0.22	5
Prop‐ *β*(Ala‐Ala)‐BY630	7.26 ± 0.09	7	7.23 ± 0.12	7	7.86 ± 0.14	5
CGP‐12177‐TMR	7.55 ± 0.15	5	7.87 ± 0.06	14	7.72 ± 0.10	5

Values show mean ± SEM obtained in *n* separate experiments. In each individual experiment triplicate determinations were made for each experimental condition. pK_i_ values were determined from *IC*_50_ values using the Cheng–Prusoff equation as described under Methods. pK_d_ values were determined from NanoBRET saturation fluorescent ligand‐binding analysis performed over 1 h or 2 h incubation. Radioligand‐binding studies were performed over 2 h incubations. BRET, bioluminescence energy transfer.

### Binding characteristics of three fluorescent *β*_1_‐adrenoceptor antagonists at the human *β*_1_‐AR using NanoBRET

**Figure 4:**
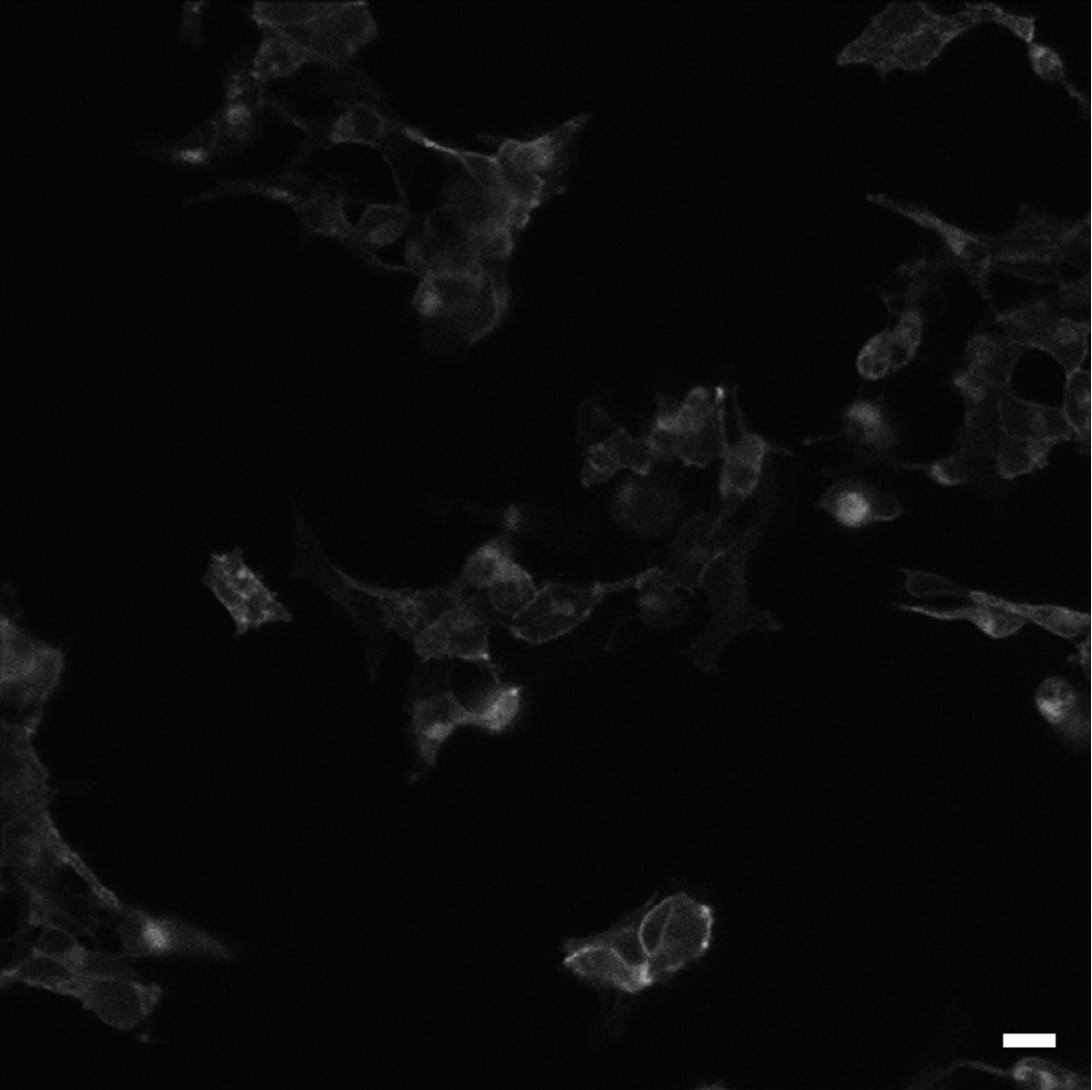
Image of HEK 293 cells transiently transfected with NL‐*β*_1_‐AR showing clear plasma membrane distribution of the fusion protein. This single 10 sec exposure image was taken immediately after the addition of 10 *μ*mol/L furimazine and is representative of five separate experiments. Scale bar is 20 *μ*m.

**Figure 5:**
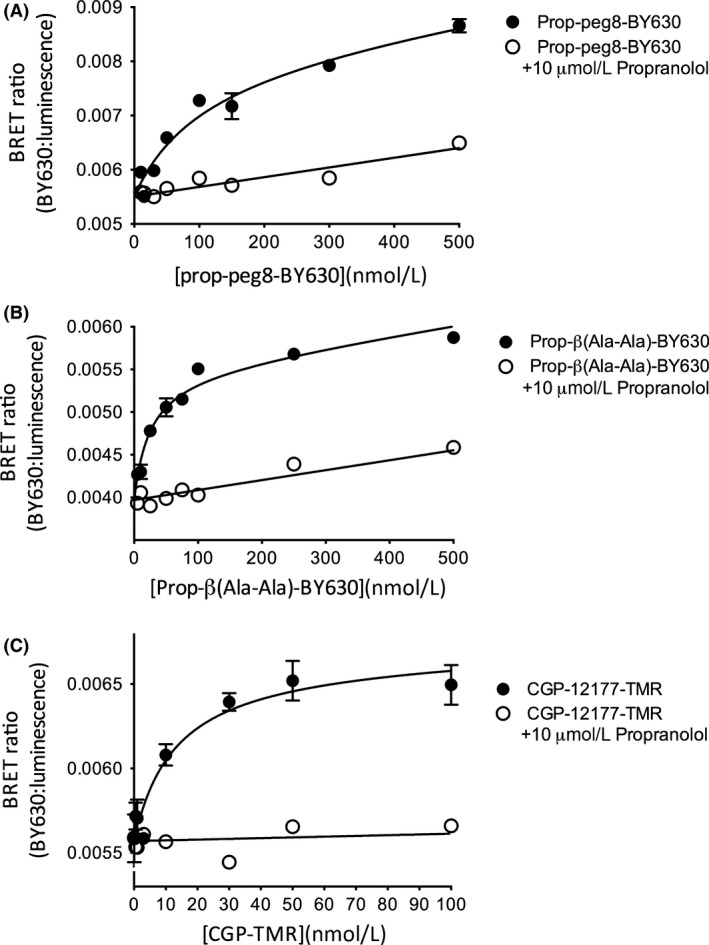
NanoBRET signal obtained from HEK 293 NL‐*β*_1_‐AR cells incubated for 1 h with increasing concentrations of (A) Propranolol‐Peg8‐BY630 (B) Propranolol‐*β*(Ala‐Ala)‐BY630, or (C) CGP‐12177‐TMR. Nonspecific binding was determined in the presence of 10 *μ*mol/L propranolol. Data points are mean ± SEM of triplicate determinations from a single experiment. These single experiments are representative of (A) eight, (B) seven, and (C) fourteen separate experiments. Where not seen, error bars are within the symbol. BRET, bioluminescence energy transfer.

**Figure 6:**
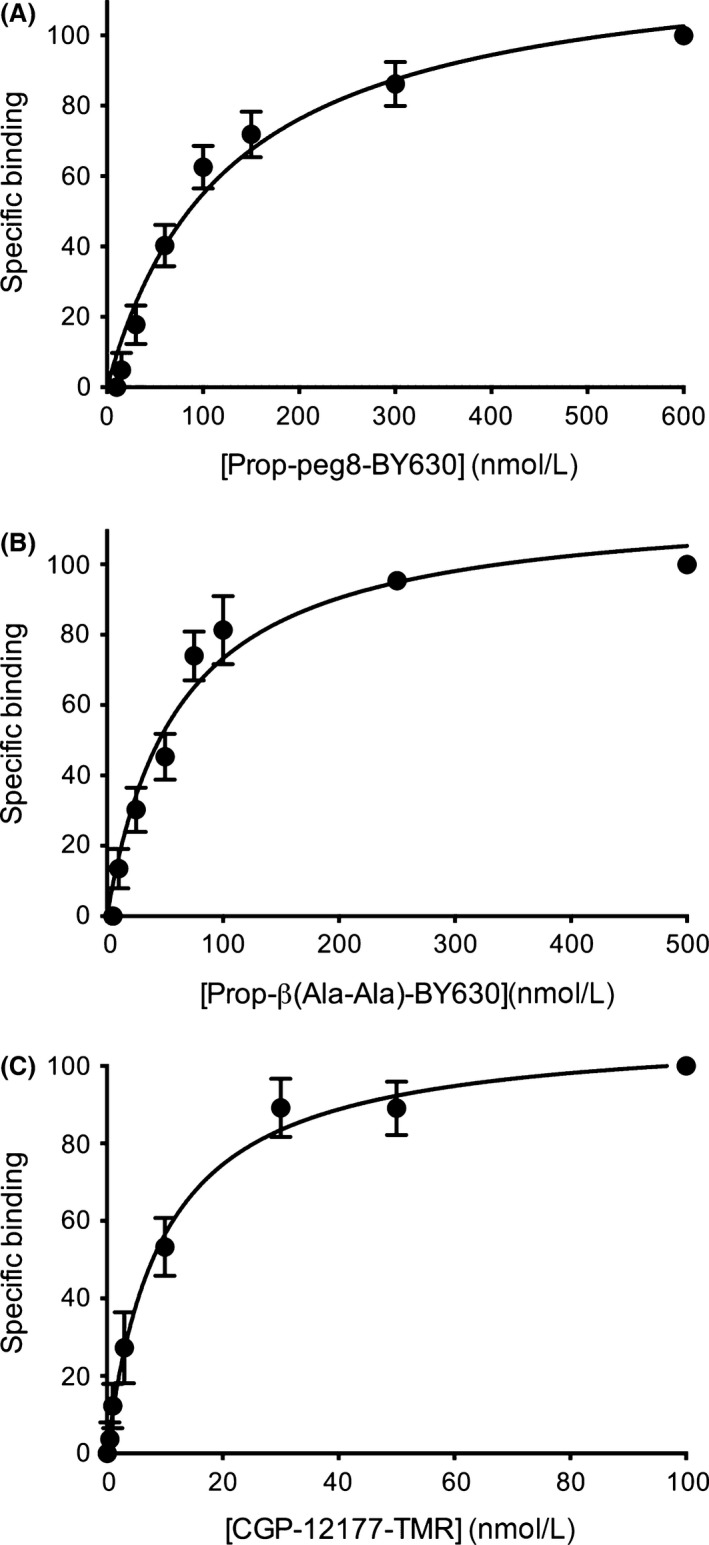
Normalized saturation‐binding curves of the specific binding of (A) Propranolol‐Peg8‐BY630 (B) Propranolol‐*β*(Ala‐Ala)‐BY630, or (C) CGP‐12177‐TMR. Data points are mean ± SEM of eight (propranolol‐Peg8‐BY630), seven (propranolol‐ *β*(Ala‐Ala)‐BY630), and fourteen (CGP‐12177‐TMR) separate experiments. Values have been normalized to the specific binding obtained at the highest concentration of each fluorescent ligand in each separate experiment. Nonspecific binding was determined in the presence of 10 *μ*mol/L propranolol and subtracted from the total binding values to obtain specific binding levels in each individual experiment. Summary data for the pK_d_ values obtained in each replicate experiment are provided in Table 2.

**Figure 7:**
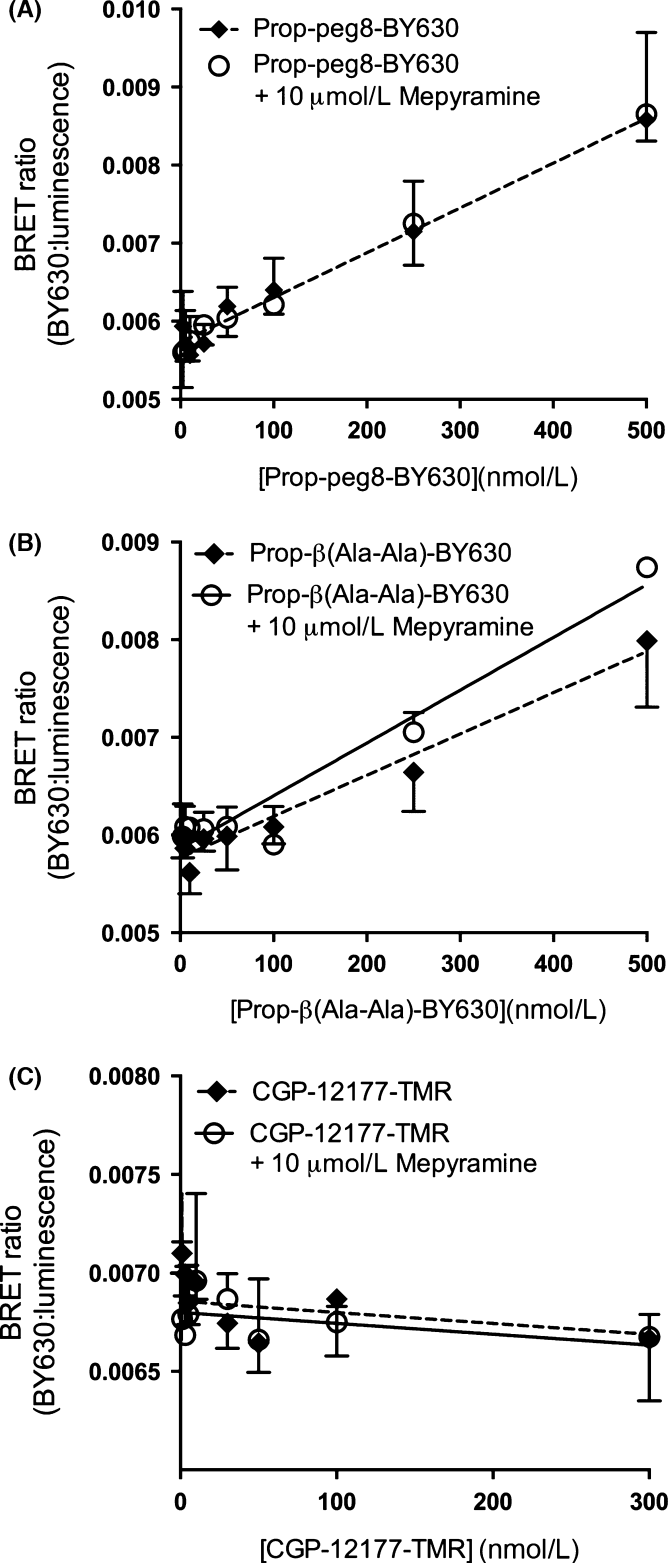
NanoBRET signal obtained from HEK 293 cells expressing an NLuc‐tagged histamine H_1_‐receptor incubated for 1 h with increasing concentrations of (A) Propranolol‐Peg8‐BY630, (B) Propranolol‐*β*(Ala‐Ala)‐BY630, (C) CGP‐12177‐TMR. Nonspecific binding was determined in the presence of 10 *μ*mol/L mepyramine. Data points are mean ± SEM from triplicate determinations in a single representative experiment. These single experiments are representative of five separate experiments. BRET, bioluminescence energy transfer.

The successful expression of the NL‐*β*_1_‐AR on the surface of HEK 293 cells is indicated by the radioligand‐binding studies with ^3^H‐CGP‐12177 (above), but also from imaging of the Nanoluc bioluminescence using TIRF microscopy (Fig. 4). In Figure 4, clear membrane‐associated luminescence can be seen at the single cell level. This cell surface expression provided the opportunity to investigate ligand binding of different fluorescent ligands using BRET (NanoBRET) to report the proximity of the fluorescent ligand to the NL‐tagged *β*_1_‐AR, as we have previously shown for other receptors (Stoddart et al. 2015). All three fluorescent ligands showed clear specific binding (as determined by BRET) to the *β*_1_‐AR (Figs. 5, 6). These data provided estimates for the *K*_D_ values of Propranolol‐Peg8‐BY630 (87.1 ± 10 nmol/L, *n* = 8; Figs. 5A, 6A), Propranolol‐ *β*(Ala‐Ala)‐BY630 (38.1 ± 12 nmol/L, *n* = 7; Figs. 5B, 6B), and CGP‐12177‐TMR (13.4 ± 2 nmol/L, *n* = 14; Fig. 5C, 6C). The latter value is very similar to the value (22.4 nmol/L; Gherbi et al. 2014) deduced with this fluorescent probe at the wild‐type *β*_1_‐AR expressed in CHO cells. To confirm that the interaction of the fluorescent ligands with the *β*_1_‐AR was receptor specific, we also evaluated their ability to generate a BRET signal in HEK 293 cell expressing an unrelated receptor, the human H_1_‐receptor with an N‐terminal NanoLuc fusion (Fig. 7). The binding of Propranolol‐Peg8‐BY630 (Fig. 7A), Propranolol‐ *β*(Ala‐Ala)‐BY630 (Fig. 7B), and CGP‐12177‐TMR (Fig. 7C) to the histamine H_1_‐receptor showed an essentially linear increase in BRET over a wide concentration range (0–500 nmol/L) consistent with nonspecific (Fig. 7A and C) or very low affinity binding (Fig. 7B).

### Probe dependence of pKi values determine for *β*_1_‐AR agonists and antagonists

**Figure 8:**
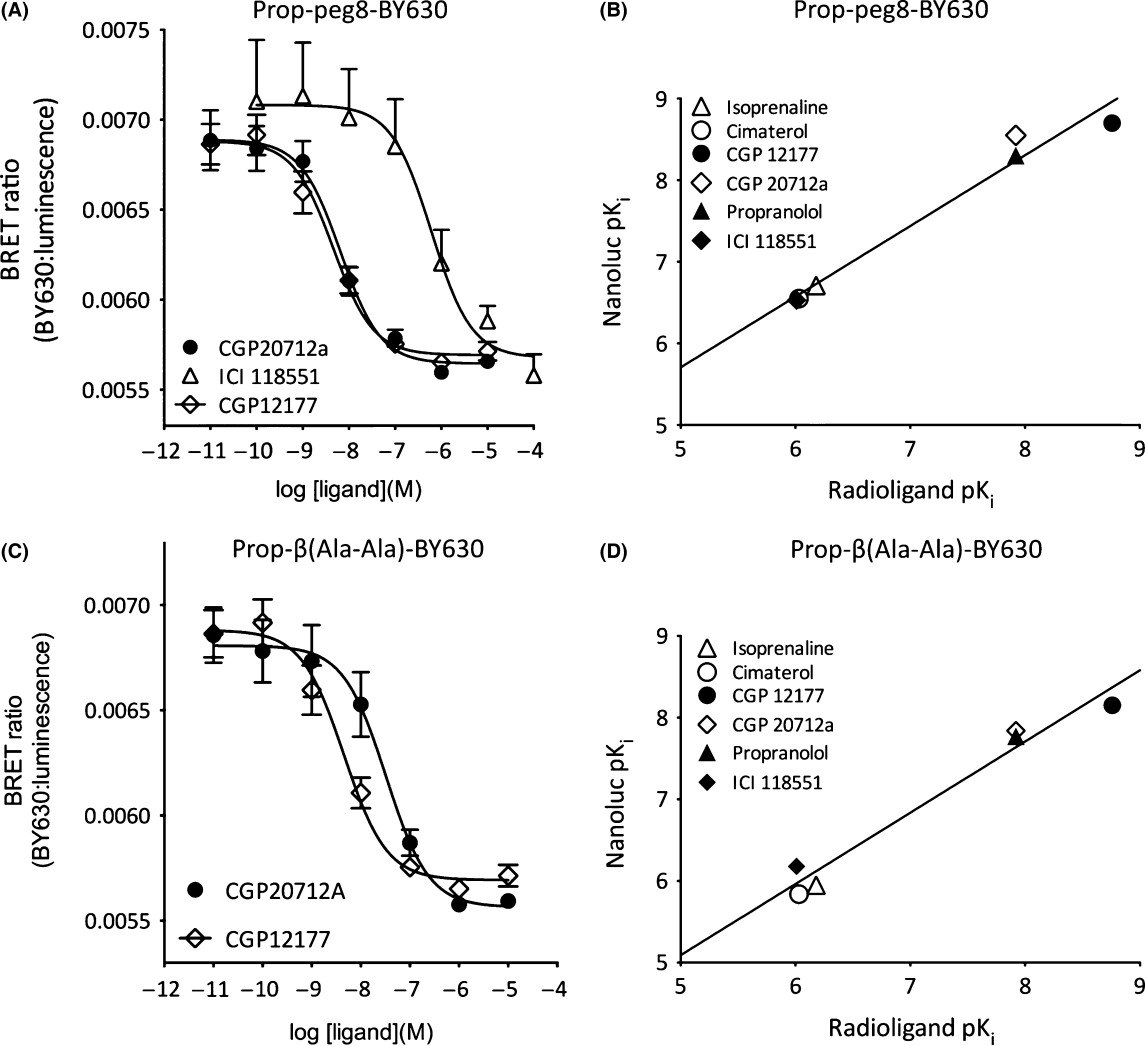
NanoBRET signal from NL‐ *β*_1_‐AR cells treated with (A–B) 100 nmol/L Propranolol‐Peg8‐BY630, (C–D) 50 nmol/L Propranolol‐ *β*(Ala‐Ala)‐BY630 and increasing concentrations of (A) CGP 20712A, ICI 118551, CGP 12177 or (C) CGP 20712A, CGP 12177. (B, D) Regression plots comparing the pK_i_ obtained from NanoBRET binding to those obtained using radioligand binding. Data points are combined mean ± SEM from (A–B) seven and (C–D) eight separate experiments. Exceptions are propranolol and ICI 118551 in both figures where data represent mean ± SEM from five separate experiments. BRET, bioluminescence energy transfer.

**Figure 9:**
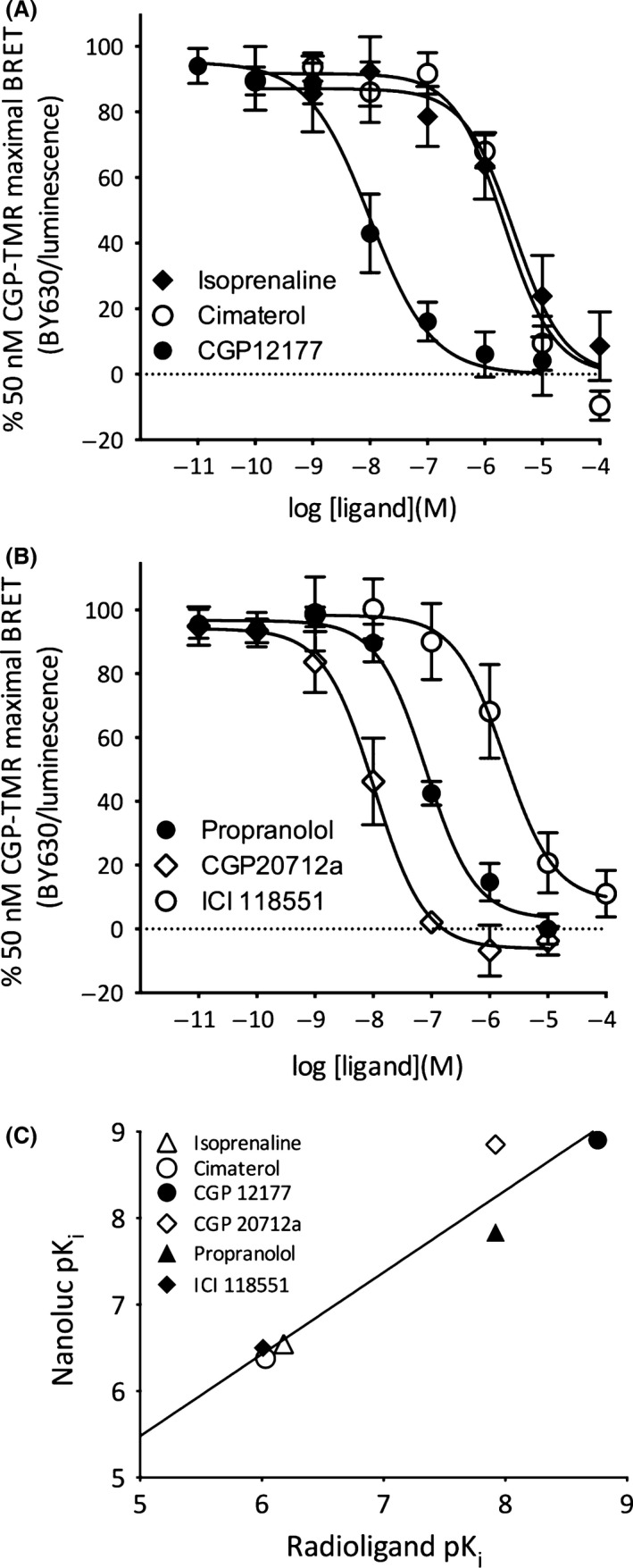
NanoBRET signal from NL‐*β*_1_‐AR cells treated with 50 nmol/L CGP‐12177‐TMR and increasing concentrations of (A) isoprenaline, cimaterol, CGP‐12177 (B) propranolol, CGP 20712A, ICI 118551. Data points are expressed as a % of the specific binding of CGP‐12177‐TMR obtained (in the absence of inhibitor) in each individual experiment and represent the mean ± SEM from five separate experiments. Nonspecific binding was determined in the presence of 10 *μ*mol/L propranolol. (C) Regression plot comparing the pK_i_ obtained from the NanoBRET experiments in (A–B) to pK_i_ obtained with radioligand‐binding studies. BRET, bioluminescence energy transfer.

**Figure 10:**
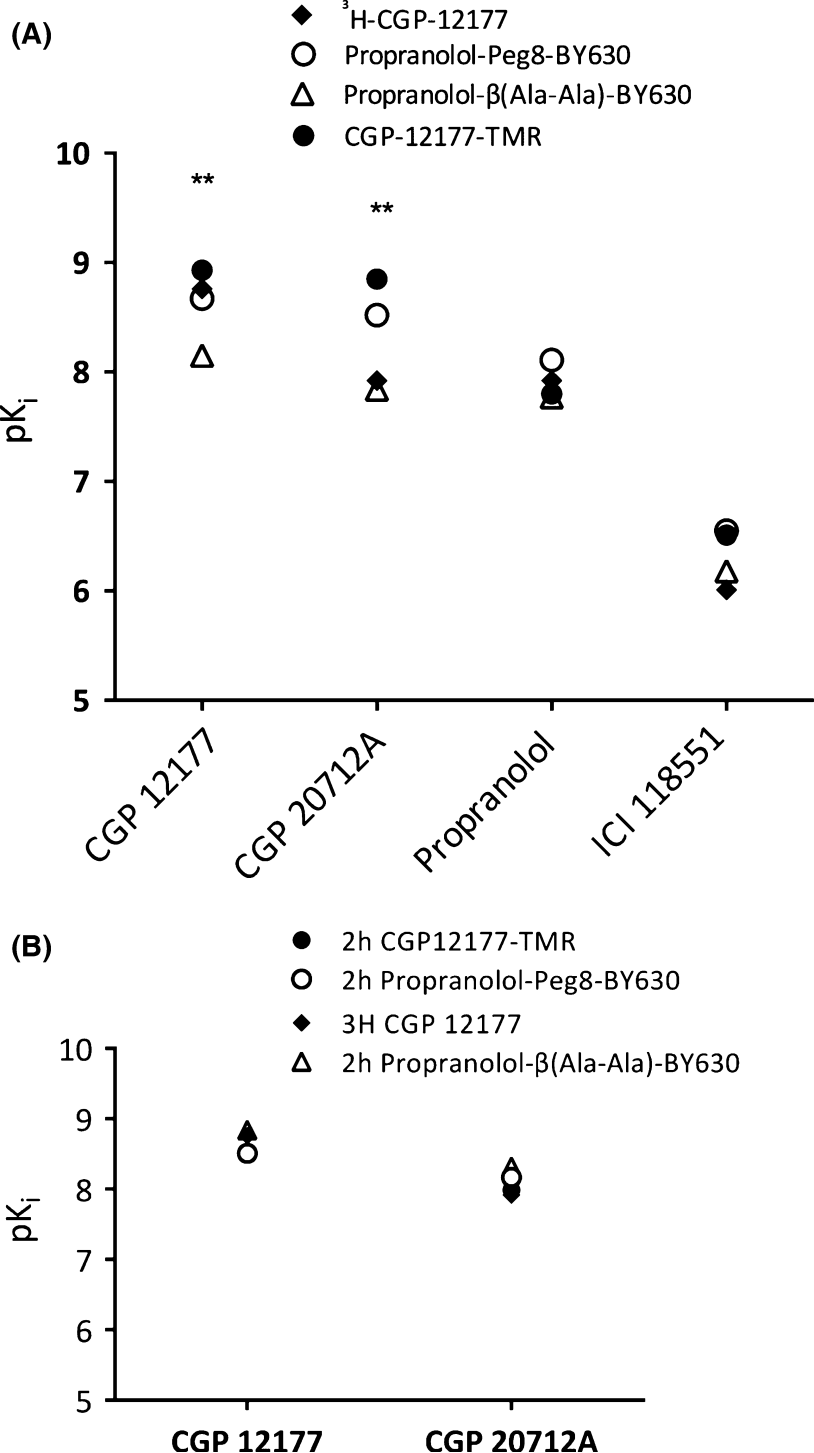
A comparison of pKi values obtained at the NL‐tagged *β*_1_‐adrenoceptor using three different fluorescent *β*_1_‐adrenoceptor ligands (Propranolol‐Peg8‐BY630, Propranolol‐*β*(Ala‐Ala)‐BY630, or CGP‐12177‐TMR) and ^3^H‐CGP 12177. Incubation with fluorescent ligands was for either 1 h (A) or 2 h (B). pK_i_ values were taken from Table 1 (A) or Table 3 (B) (see tables for SEMs and *n* numbers). **,*pK_i_ values obtained for competing ligand significantly differ between fluorescent/radioactive probes (**P < 0.001; One‐way ANOVA).

The availability of three different fluorescent ligands for the *β*_1_‐AR provided the opportunity to investigate whether competition‐binding experiments exhibited probe dependence, and the extent to which the values obtained for pK_i_ differed from those obtained from radioligand‐binding experiments. Competition‐binding experiments were initially undertaken with the two fluorescent propranolol derivatives that essentially only differ in the length and composition of the linker between propranolol and the BODIPY 630/650 fluorophore (Fig. 8). In both cases, there was a reasonable agreement with the values obtained from radioligand‐binding experiments with ^3^H‐CGP 12177 (Fig. 8B and D). The slope of the linear regression lines in Figures 8B and D were 0.87 ± 0.08 (*R* ^2 ^= 0.97; *y* intercept = 1.38 ± 0.56) and 0.87 ± 0.08 (*R* ^*2*^ = 0.97; *y* intercept = 0.73 ± 0.60), respectively. However, a comparison of the pK_i_ values obtained with the two fluorescent propranolol derivatives revealed a trend to higher values obtained with Propranolol‐Peg8‐BY630 relative to Propranolol‐*β*(Ala‐Ala)‐BY630 (Table 1). Comparable higher pK_i_ values were also obtained with CGP‐12177‐TMR (Fig. 9; Table 1). However, although the correlation between pK_i_ values obtained with ^3^H‐CGP 12177 and CGP‐12177‐TMR was similar to the other comparisons (Fig. 9C; 0.95 ± 0.14; *y* intercept 0.74 ± 1.00; *R* ^*2*^ = 0.92) the values obtained with CGP 20712A differed by an order of magnitude. A comparison of the pK_i_ values obtained for each competing antagonist ligand suggested that there were significant probe‐dependent differences between them (Fig. 10). In the case of CGP20712A and CGP 12177, the differences were highly significant (*P* < 0.001; one‐way ANOVA; Fig. 10A).

### Impact of incubation time

One clear difference between the experimental conditions used for fluorescent ligand‐binding (1 h incubation) and radioligand‐binding (2 h incubation) studies was incubation time. The initial choice of 1 h for incubation with fluorescent ligands was to limit the potential for uptake of the more lipophilic ligands into the cells, which has been previously observed to increase the nonspecific binding determined in fluorescence intensity measurements (Baker et al. 2011; Gherbi et al. 2014). To explore the potential for the shorter incubation time to be a confounding factor due to differences in the ligand‐binding kinetics of the ligands used (both fluorescent and competing ligands), we repeated the fluorescent ligand experiments with CGP 20712A and CGP 12177 as competing drugs over 2 h incubations. Analysis of these data indicated that there were no significant differences between pK_i_ values obtained with different fluorescent ligands or ^3^H‐CGP12177 following 2 h incubation (Table 3, Fig. 10B). Here, the *IC*_50_ values for CGP 12177 and CGP 20712A were corrected for the presence of the fluorescent ligand using the Cheng–Prusoff equation and the pK_d_ values for the three fluorescent ligands obtained from saturation analysis at 2 h incubation (Table 2).

**Table 3:** Binding affinities of competing ligands determined from inhibition of the specific binding of a 2‐h incubation with 0.9–2.5 nmol/L ^3^H‐CGP 12177, 100 nmol/L Propranolol‐Peg8‐BY630, 50 nmol/L Propranolol‐ *β*(Ala‐Ala)‐BY630, or 50 nmol/L CGP‐12177‐TMR in HEK 293 cells expressing a NanoLuc‐tagged human *β*_1_‐adrenoceptor

	^3^H‐CGP‐12177		Propranolol‐Peg8‐BY630		Propranolol‐*β*(Ala‐Ala)‐BY630		CGP‐12177‐TMR	
	pKi	*n*	pKi	*n*	pKi	*n*	pKi	*n*
CGP 12177	8.76 ± 0.09	5	8.51 ± 0.13	9	8.84 ± 0.14	5	8.51 ± 0.10	9
CGP 20712A	7.92 ± 0.11	5	8.17 ± 0.16	7	8.32 ± 0.16	5	7.99 ± 0.10	8

Values show mean ± SEM obtained in *n* separate experiments. In each individual experiment triplicate determinations were made for each experimental condition. pK_i_ values were determined from *IC*_50_ values using the Cheng–Prusoff equation as described under Methods. pK_i_ values obtained of competing ligand do not significantly differ between fluorescent/radioactive probes (One‐way ANOVA).

## Discussion and Conclusions

This study has confirmed that the recently described NanoBRET proximity assay for the study of ligand binding to cell surface GPCRs (Stoddart et al. 2015) can be applied to the human *β*_1_‐adrenoceptor expressed in HEK 293 cells. The presence of the NanoLuc tag on the N‐terminus of the *β*_1_‐adrenoceptor did not prevent a good level of cell surface expression of the receptor, as determined by both single cell bioluminescence imaging of the NanoLuc tag and whole‐cell radioligand‐binding studies with ^3^H‐CGP 12177 (circa 1500 fmol. mg protein^−1^). Radioligand‐binding studies with ^3^H‐CGP 12177 also confirmed that the NLuc‐ *β*_1_‐adrenoceptor had high affinity for the *β*_1_‐adrenoceptor‐selective ligand CGP 20712A and low affinity for the *β*_2_‐adrenceptor antagonist ICI 118551.

Three different fluorescent *β*‐AR ligands were used to investigate binding to the *β*_1_‐AR in this study: (1) Propranolol‐Peg8‐BY630 (Baker et al. 2011); (2) Propranolol‐ *β*(Ala‐Ala)‐BY630 (Stoddart et al. 2015), and (3) CGP‐12177‐TMR (Gherbi et al. 2014). Only one of these has been previously used in a NanoBRET ligand‐binding assay, and that was to study binding to a NanoLuc‐tagged human *β*_2_‐adrenoceptor (Stoddart et al. 2015). All three ligands showed good receptor‐specific binding to the human *β*_1_‐adrenoceptor in HEK 293 cells. Their rank order of affinity (*K*_D_ values) was: CGP‐12177‐TMR (13.4 nmol/L), propranolol‐ *β*(Ala‐Ala)‐BY630 (38 nmol/L), and propranolol‐Peg8‐BY630 (87.1 nmol/L). Interestingly, this proximity‐based assay allowed ligand binding to be monitored over a wide concentration range and nonspecific binding was not excessive. Nonspecific binding was greatest for propranolol‐ *β*(Ala‐Ala)‐BY630 and propranolol‐Peg8‐BY630. This is likely to be a consequence of partitioning in the membrane of these more lipophilic ligands in close proximity to the NLuc tag on the *β*_1_‐adrenoceptor.

To investigate further whether these fluorescent ligands could generate a BRET signal with the N‐terminal NLuc‐tag of the *β*_1_‐adrenoceptor from nonspecific partitioning in the adjacent membrane (i.e., in close proximity to the receptor), we studied an unrelated NLuc‐tagged GPCR, namely the histamine H_1_‐receptor. Interestingly, both propranolol‐ *β*(Ala‐Ala)‐BY630 and propranolol‐Peg8‐BY630 generated a linear concentration‐dependent increase in energy transfer from the NLuc of the H_1_‐receptor to the fluorescent ligand that was consistent with some component of the BRET signal being due to the fluorescent ligand in the adjacent plasma membrane. In contrast, when CGP‐12177‐TMR was used as the fluorescent ligand there was no concentration‐dependent increase in nonspecific binding. In the case of both propranolol‐Peg8‐BY630 and CGP‐12177‐TMR, the binding obtained was not inhibited by 10 *μ*mol/L mepyramine, while any effect on propranolol‐ *β*(Ala‐Ala)‐BY630 was marginal, confirming that it was predominately nonspecific in nature.

The specific binding of each fluorescent ligand to the NL‐*β*_1_‐adrenoceptor was antagonized by a range of antagonists in a manner consistent with that expected of a specific *β*1‐adrenoceptor interaction. However, closer inspection of the pK_i_ values obtained for individual competing antagonists indicated that some of them varied significantly depending on the particular fluorescent ligand used as the probe (Fig. 10A). This was particularly the case for CGP 12177 and CGP 20712a (Fig. 10A). One clear difference between the experimental conditions used for fluorescent ligand binding and those for radioligand‐binding studies was incubation time. The initial choice of 1 h for incubation with fluorescent ligands was designed to limit the potential for uptake of the more lipophilic ligands into the cells, which tends to increase the nonspecific binding determined in fluorescence intensity measurements (Baker et al. 2011; Gherbi et al. 2014; Rose et al. 2012). However, if the incubation times used for the various assays are not sufficiently long to achieve equilibrium, then over‐ or under‐estimates of pK_i_ values for certain competing ligands may be obtained (Motulsky and Mahan 1984). This may be compounded if the probe or competing drugs are lipophilic and their rate of achieving equilibrium is delayed by membrane‐binding interactions (Sykes et al. 2014; Vauquelin and Charlton 2010; Vauquelin 2010).

Extending the incubation time for the NanoBRET competition assay to 2 h provided consistent measurements of ligand binding with all three fluorescent ligands. Furthermore, in keeping with the above hypothesis, extension of the fluorescent ligand‐binding incubation time from 1 h to 2 h removed any significant probe‐dependent differences in the observed pK_i_ values obtained (Fig. 10B). These data suggest that at the concentrations of fluorescent ligand employed here there is no evidence of probe dependence. The data obtained at the two incubation times also emphasize the importance of ensuring that both the fluorescent and competing ligands are in true equilibrium before interpretations regarding probe dependence can be made.

In summary, we have shown here that a NanoBRET proximity assay can be used to undertake a detailed characterization of the ligand‐binding characteristics of three different fluorescent ligands at the human *β*_1_‐adrenoceptor expressed in living HEK 293 cells. Provided that the incubation time was sufficiently long to achieve equilibrium, the pK_i_ values obtained for four different *β*‐adrenoceptor antagonists did not provide any evidence for probe dependence at the human *β*1‐adrenoceptor. Further studies will be required using mutagenesis and kinetic approaches to determine whether there is allosterism across dimer interfaces involving the *β*_1_‐adrenoceptor in HEK 293 cells similar to that observed previously in CHO‐K1 cells (Gherbi et al. 2015).

## Author Contributions

Soave, Stoddart, Brown, Woolard, and Hill participated in research design. Soave conducted experiments. Soave and Hill performed data analysis. Soave, Brown, Woolard, and Hill wrote or contributed to the writing of the manuscript.

## Disclosures

The authors declare no conflict of interest.
